# School Children and Their Industries

**Published:** 1899-05-13

**Authors:** 


### School Children and their Industries.

Since we wrote last week upon the physiological aspects of education a somewhat lurid light has been thrown, by a highly instructive debate in the House of Commons, upon conditions of industry in relation to school children, which are opposed not only to physiology but also to common sense, and which require revision in the interests of the health of the community as much as in the interests of its intellectual development. Sir John Gorst, in introducing last year the estimates for the Education Department, expressed his belief that a large number of children attending school were also employed in labour to the full measure of their powers, and were prevented by fatigue from any profitable pursuit of learning. In discharging the same duty last week, he informed the House that an inquiry into this question had been instituted by the Department, and he was able to confirm, by precise facts and figures, a belief which had previously rested in some measure upon conjecture. It appears that 144,000 children, who are full time scholars, are also engaged as labourers for wages or profit, and that there are many more of whom the same thing might be said, but who, from one reason and another, have not been included in the return. As a specimen of the cases cited, it is said that "one boy begins work for his father as early as three a.m., and works again in the evening as late as nine p.m. *He often goes to sleep during morning school from sheer weariness.*" A little boy of six is engaged in peeling onions twenty hours a week for a wage of eightpence. Another delivers milk during twenty-eight hours a week. Another of the same age is engaged in turning hose for twenty hours a week; and the "champion boy," who is under six years of age, works in a brickfield at brick-making, and earns a wage of 3s. 6d. a week. Girls, even at the age of six, are engaged for hire as nurses to younger children. Some of the boys are regularly employed to call labourers who have to rise early. One of these boys "rises between three and four every morning, starts out at half-past four a.m. to wake up twenty-five working men, and returns from his rounds about half-past five, but does not go to bed again, as at six o'clock he has to go round as a 'newspaper boy' till nine o'clock, when he comes to school. He is a very regular boy, but is often half asleep, especially on the afternoons of hot days." Such are but a few of the examples which Sir John cited to the House, and he promised the early publication of the report from which they were derived.

It is a familiar saying that the horse, although the most honest of animals, possesses a peculiar power of promoting the development of rascality in men who are much associated with him; and in like manner the general question of education, than which there can be few of greater national importance, appears to act upon a deliberative assembly chiefly as a stimulus to the growth of the *odium theologicum.* Mr. Bryce, in the course of an admirable speech, told the House of Commons that the question before them related to the investment of money "which was to bear fruit in an active, useful, energetic generation thirty years hence"; but, even with this consideration thus rendered prominent, the representatives of the people wandered off into a discussion about catechisms. It is manifest, not only that the money expended upon the education of the 144,000 children referred to, and of the many others known to be subject to similar conditions, must be wholly thrown away, but also that the disclosures concerning child labour of (as was aptly said by a school manager) "thousands of little white slaves" have a very important bearing upon questions of public health. Every parent knows that one of the first essentials for a young child is a sufficient period of sound and refreshing sleep; and it cannot be doubted that the "little white slaves," deprived of this by the greed of their parents, probably badly treated also in other ways, and called upon for efforts to learn in spite of physical exhaustion, would be the earliest victims of any form of contagion which might be introduced into their school, and which, as we all know, schools are such important agents in diffusing among the juvenile population. It is one of the worst characteristics of the lower strata of the labouring classes that they are not only willing, but desirous, to live upon the earnings of their young children; a feeling which may be part of the evil heritage left behind by that old system of Poor Law under which early marriages were encouraged, and every additional child was regarded as a reason for increased parochial relief in aid of agricultural or other wages. Even now, in the chief localities of the industries in which child labour is employed, parents resort to all kinds of tricks in order to evade the Factory Acts, and to represent their children as older than they are, even registering successive children under the same Christian name, and appealing to the registration of the first as evidence of the ages of subsequent ones. It seems certain that, in the interests of health, no less than in those of education, it will be necessary soon to submit the whole question of child labour to the consideration of Parliament, and to subject it to control of a more stringent kind than has ever hitherto been enforced.

